# Temporal Variability of Pesticide Concentrations in Homes and Implications for Attenuation Bias in Epidemiologic Studies

**DOI:** 10.1289/ehp.1205811

**Published:** 2013-03-05

**Authors:** Nicole C. Deziel, Mary H. Ward, Erin M. Bell, Todd P. Whitehead, Robert B. Gunier, Melissa C. Friesen, John R. Nuckols

**Affiliations:** 1 Occupational and Environmental Epidemiology Branch, Division of Cancer Epidemiology and Genetics, National Cancer Institute, National Institutes of Health, Department of Health and Human Services, Bethesda, Maryland, USA; 2 Department of Environmental Health Sciences, School of Public Health, University at Albany, Rensselaer, New York, USA; 3 Division of Environmental Health Sciences, School of Public Health, University of California, Berkeley, Berkeley, California, USA; 4 Department of Environmental and Radiological Health Sciences, Colorado State University, Fort Collins, Colorado, USA

**Keywords:** dust, environmental exposure, pesticides, reliability

## Abstract

Background: Residential pesticide exposure has been linked to adverse health outcomes in adults and children. High-quality exposure estimates are critical for confirming these associations. Past epidemiologic studies have used one measurement of pesticide concentrations in carpet dust to characterize an individual’s average long-term exposure. If concentrations vary over time, this approach could substantially misclassify exposure and attenuate risk estimates.

Objectives: We assessed the repeatability of pesticide concentrations in carpet dust samples and the potential attenuation bias in epidemiologic studies relying on one sample.

Methods: We collected repeated carpet dust samples (median = 3; range, 1–7) from 21 homes in Fresno County, California, during 2003–2005. Dust was analyzed for 13 pesticides using gas chromatography–mass spectrometry. We used mixed-effects models to estimate between- and within-home variance. For each pesticide, we computed intraclass correlation coefficients (ICCs) and the estimated attenuation of regression coefficients in a hypothetical case–control study collecting a single dust sample.

Results: The median ICC was 0.73 (range, 0.37–0.95), demonstrating higher between-home than within-home variability for most pesticides. The expected magnitude of attenuation bias associated with using a single dust sample was estimated to be ≤ 30% for 7 of the 13 compounds evaluated.

Conclusions: For several pesticides studied, use of one dust sample to represent an exposure period of approximately 2 years would not be expected to substantially attenuate odds ratios. Further study is needed to determine if our findings hold for longer exposure periods and for other pesticides.

Residential exposure to pesticides has been linked to several adverse health outcomes, including adult cancers, such as non-Hodgkin lymphoma (Colt et al. 2006; Ward et al. 2009) and prostate cancer (Cockburn et al. 2011); childhood cancers, such as non-Hodgkin lymphoma, leukemia, and brain cancer (Infante-Rivard and Weichenthal 2007; Metayer and Buffler 2008; Van Maele-Fabry et al. 2011); and neurodevelopmental deficits (Bouchard et al. 2011; Engel et al. 2011; Rauh et al. 2011; Rosas and Eskenazi 2008). In epidemiologic studies of cancer, self-reported pesticide use is typically used to estimate residential pesticide exposure because of its low cost and participant burden (Ritz and Rull 2008). Limitations include potentially inaccurate or differential participant recall and lack of information on specific active ingredients (Colt et al. 2004; Jurewicz and Hanke 2006). Studies of outcomes with shorter latency periods than cancer, such as neurotoxicity, have used biological measurements of pesticides in blood and urine, which are independent of recall. However, urinary pesticide metabolites are generally limited by short half-lives, large temporal variability, and lack of specificity for parent compounds that may differ in toxicity (Barr and Angerer 2006; Sams and Jones 2011). Measurements of pesticides in blood tend to have high specificity, but low frequency of detection in the general population (Barr and Angerer 2006).

Measurement of pesticides in indoor dust may be a useful indicator of long-term residential pesticide exposure because the chemicals resist degradation due to limited sunlight and microbial activity, lack of moisture, and other factors (Lewis et al. 1994; Simcox et al. 1995). Strengths of this approach include the ability to analyze the dust samples for numerous pesticide active ingredients and the lack of reliance on participant self-report. A possible shortcoming is that one sample may not be representative of average residential pesticide levels or of past exposures during critical time periods (Egeghy et al. 2005; Rappaport 1991; Whitehead et al. 2012).

Despite the advantages of pesticide measurements in carpet dust, few epidemiologic studies have incorporated such measurements to estimate residential exposure to pesticides. In the studies that have used this approach, one carpet dust sample per participant was collected, analyzed for pesticide concentrations, and used as an estimate of an individual’s chronic exposure (Colt et al. 2006; Hartge et al. 2005; Ward et al. 2009). Because concentrations of pesticides within a home may change over time as a result of pesticide use, human activities, outdoor sources, translocation, or removal mechanisms (Stout and Mason 2003), using a single measure to represent an individual’s average, long-term exposure could potentially result in measurement error and misclassification of exposure of study participants, potentially attenuating risk estimates.

In the present study, we analyzed repeated carpet dust samples for concentrations of common home and garden pesticides over an approximately 2-year period to evaluate whether a single carpet dust sample is representative of multiple samples. Using a variance components analysis, we characterized the potential impact of attenuation bias in epidemiologic studies that rely on a single sample as a surrogate of long-term average carpet dust concentrations over this time period. We also evaluated predictive factors that explained variability in pesticide concentrations between and within the study homes.

## Methods

*Study population and design*. We recruited 21 residents of Fresno County, California, an agricultural area in the Central Valley, for the Fresno Agricultural Pesticide Study (Gunier et al. 2011). Eligibility criteria included having at least 25% of the land area within 500 m of the residence in crop production and at least 24 ft^2^ of carpets or rugs in the home for ≥ 1 year. Homes were ineligible for sampling if any resident had worked in the fields of a commercial farm within the preceding 6 months. The study protocol received approval from the institutional review boards at Colorado State and Fresno State Universities and the National Cancer Institute, and all participants provided written informed consent. We conducted 1–7 data-collection visits (median, 3 visits) per residence between April 2003 and November 2005 for a total of 68 visits. The time between visits ranged from 3 to 15 months (median, 5 months), and the total follow-up time across residences with > 1 visit ranged from 2.5 to 28 months (median, 24 months).

*Housing, pest treatment, and sampling characteristics.* At the first visit to a residence, a trained interviewer collected information about household characteristics, including the presence of cats or dogs and whether they spent > 1 hr outside/day, whether family members routinely removed their shoes before entering the home, whether any family members held a pesticide-related occupation in the preceding 12 months (e.g., farmer, pesticide handler), and when the home was built (approximate decades). Residence age was grouped into three categories (< 1970, 1970–1989, ≥ 1990) that ensured a reasonable distribution of homes and reflected changes in pesticide regulations [i.e., the U.S. Environmental Protection Agency (EPA) was founded in 1970 and soon afterward began regulating pesticides, such as banning all uses of chlordane in 1988]. We combined the questions about cats and dogs into a single variable (no cat or dog, dog only, both), because they were correlated and no one had reported having only a cat. At the first visit, participants were asked whether a member of the household or a pest-control professional had treated for pests during the previous 12 months. Pest treatments included treating for fleas/ticks, ants/flies/roaches, other indoor pests, bees/wasps/hornets, and lawn/garden pests/weeds as well as professional indoor treatments, professional outdoor treatments, and professional lawn/yard treatments. The lawn/yard treatments included treating for insects and/or weeds. At each subsequent visit, participants were asked whether any of these types of pest treatments had occurred since the previous visit. We combined professional indoor and outdoor pesticide treatments into a single variable (professional outdoor, both professional indoor and professional outdoor treatments, or neither) because they were highly correlated, and no one had reported professional indoor treatment only. Sampling characteristics were recorded at each visit, and included the room sampled, whether the room was a throughway, age of the carpet, and date. We grouped carpet age (< 4 years, 4–10 years, ≥ 10 years) based on the sample distribution. We evaluated the trend in concentrations over time by calculating the difference (in months) between the first visit and subsequent visits (“months after first study visit”). We also explored season, month of sampling, and days between visits, but we considered months after first study visit as a time-related variable for all statistical models because it showed the most consistent relationship with pesticide concentrations in exploratory analyses.

*Estimates of agricultural and public land pesticide applications*. Although our emphasis was on home and garden pesticides, most pesticides we studied had both residential and nonresidential uses. Therefore, we considered the contribution of outdoor agricultural and public land applications to variability in indoor pesticide concentrations using a previously developed metric designed to estimate the density (mass/unit area) of pesticide active ingredients applied within a user-specified buffer zone (Gunier et al. 2011; Nuckols et al. 2007). The metric was based on the California Pesticide Use Reporting Database (CPUR), which includes the date, location, amount, and crop treated for pesticides applied from 1990 onward and is reported per U.S. Public Land Survey sections (~ 1 mi^2^) (California Department of Pesticide Regulation 2000). We computed the metric for the 13 pesticides measured in our study except for 3 that had no or limited agricultural/public land applications in California during the study period (i.e., chlordane, methoxychlor, propoxur). We included pesticide applications in sections within 1,250 m of study residences. We selected 1,250 m because we previously observed that pesticide applications within this distance were more strongly associated with pesticide concentrations in house dust compared with applications within shorter distances (Gunier et al. 2011). For the first visit, we estimated the density of agricultural/public land pesticide applications over the previous year. For subsequent visits, we computed the metric for the time since the last visit. Most (77%) of the pesticides (by weight) applied within sections located ≤ 1,250 m of homes were to crops, with the remaining 23% applied to public areas such as parks, ditches, and roadside and railroad right-of-ways. We evaluated an additional density metric (Gunier et al. 2011; Nuckols et al. 2007), which accounted for the location of crops within the buffer zone. The two metrics yielded similar results in the statistical models; therefore, only one metric [the “CPUR metric” (density of pesticide use in kilograms per square kilometer using the CPUR database)] is presented here.

*Dust sample collection*. As previously described (Colt et al. 2008), at each visit we collected approximately 10-mL dust samples in Teflon bottles using the high volume surface sampler vacuum. Briefly, the interviewer selected a room from the side of the home facing agricultural fields that contained carpets or rugs measuring at least 24 ft^2^. Initially, an approximate 4 ft × 6 ft area was vacuumed. Up to three areas were vacuumed to obtain a sufficient volume of dust. Subsequent samples were taken from the same room. Eighty-one percent of samples were collected from the living room or family room. Samples were transported on ice to the laboratory. Vacuums were cleaned with isopropanol between homes.

*Laboratory analysis*. We shipped samples to the Battelle Memorial Institute (Columbus, OH), where they were stored at –20°C until processing and analysis, as described previously (Colt et al. 2008). Dust samples were sieved (150 μm), spiked with ^13^C-labeled surrogate recovery standards (SRSs), and extracted with a 1:1 vol:vol solution of hexane:acetone. We analyzed samples for 13 home and garden pesticides using gas chromatography–mass spectrometry in the multiple ion detection mode. We achieved quantification with an 8-point calibration curve, ranging from 2 to 750 ng/mL for analytes and 10 to 300 ng/mL for SRSs, and included an instrument blank in each sample set. The target analytes were carbaryl, propoxur, chlordane (α- and γ- isomers), methoxychlor, chlorpyrifos, diazinon, cyfluthrin (four chromatically resolved isomers), cypermethrin (four chromatically resolved isomers), permethrin (*cis*- and *trans*- isomers), piperonyl butoxide, dacthal, simazine, and trifluralin. These insecticides and herbicides represent a range of pesticide classes: carbamates, organochlorines, organophosphates, and pyrethroids in addition to a pesticide synergist, a chlorinated benzoic acid, a triazine, and a dintroaniline. Because of the extraction method used, we were not able to measure some of the more common residential herbicides, including 2,4-dichlorophenoxyacetic acid, dicamba, and glyphosate. Quality control samples in each batch included an instrument blank, sample duplicates, and duplicate laboratory spikes. Duplicate samples had average relative percent differences of 10–30%. Mean sample recoveries for spiked samples ranged from 85 to 118%; SRS recoveries averaged 82–111%.

*Statistical analysis*. We conducted all analyses in SAS version 9.3 (SAS Institute Inc., Cary, NC). Natural log–transformed pesticide concentrations were used in all analyses; concentrations of all isomers of a pesticide were summed. We imputed values below the limit of detection (LOD) using a maximum likelihood procedure that assumed a lognormal distribution defined by the distribution of the measurements above the LOD (Lubin et al. 2004). The imputation was repeated five times.

For each pesticide, the between-home (σ^2^_BW_) and within-home (σ^2^_WI_) variance components were calculated using regression models that included home as a random effect (“null models”) (Equation 1):

ln(*Y_ij_*) *=* μ*_y_ + b_i_ +* ε*_ij_,* [1]

where *i* represents the number of homes; *j* is the number of repeated measurements; ln(*Y_ij_*) is the natural log–transformed pesticide concentration for the *i*th home for the *j*th repeated measurement; μ*_y_* is the mean (logged) pesticide concentration for the population; *b_i_* is the random effect for *i*th home; and ε*_ij_* is the residual error associated with the *i*th home for the *j*th repeated measure. We assumed that *b_i_* and ε*_ij_* were normally distributed and independent, with means of 0 and variances of σ*^^2^^*_BW_ and σ*^^2^^*_WI_, respectively. Models were constructed with PROC MIXED using a restricted maximum likelihood estimation procedure and assuming a uniform covariance structure. We fit each pesticide’s null model five times—once for each of the five data sets with imputed values below the LOD—and combined the results using PROC MIANALYZE (Lubin et al. 2004; Rubin and Schenker 1991). The variance components from the null models were used to calculate the intraclass correlation coefficient (ICC) (Equation 2):

ICC = σ*^2^*_BW_*/*(σ*^2^*_BW_ + σ*^2^*_WI_). [2]

We computed the expected attenuation of odds ratios for a hypothetical case–control study, assuming that the logistic model (Equation 3) describes the odds of disease associated with concentration of a pesticide in dust:

logit (Z*_i_*) = ln[Z*_i_/*(Z*_i_* – 1)] = β_0_ + β_1_*Y^^—^^_i_*, [3]

where *Z_i_* represents the disease status (1 or 0) of an individual in the *i*th household, *Y*^–^*_i_* is the mean pesticide concentration for the *i*th home, and β_1_ is the logistic regression coefficient [where the odds ratio = exp(β_1_)]. The observed value of the logistic regression coefficient β_1_*_,_*_obs_ is related to the true regression coefficient β_1,true_ as shown in Equation 4:


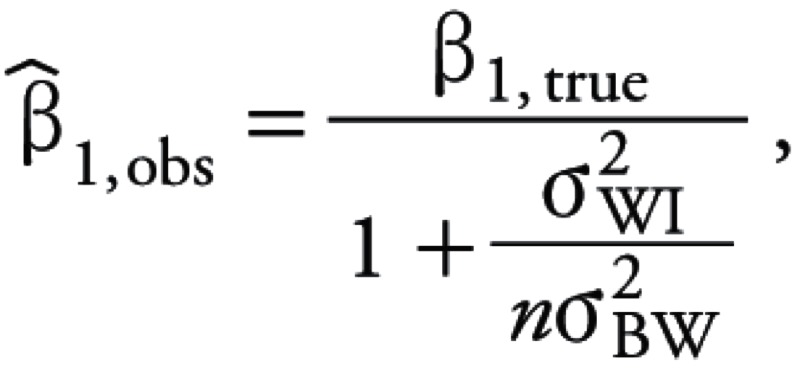
[4]

where *n* is the number of repeated samples (Cochran 1968; Whitehead et al. 2012). We defined the attenuation bias (Equation 5) as the normalized difference between the observed and true regression coefficients (Whitehead et al. 2012):


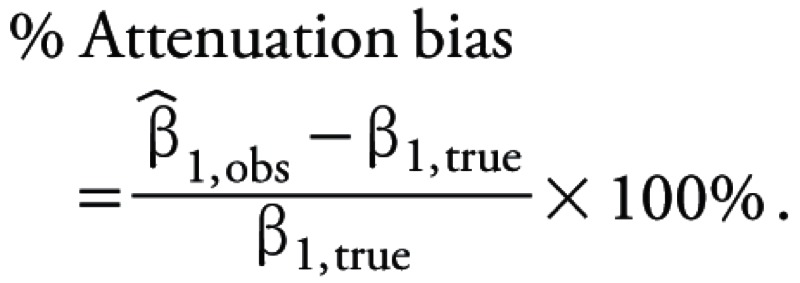
[5]

To illustrate the impact of the attenuation bias on a hypothetical odds ratio when a single measurement is used to represent average exposure, we calculated the observed odds ratio (OR_obs_) assuming a true odds ratio (OR_true_) of 2.0 (Equation 6) (Cochran 1968):

OR_obs_ *=* OR_true_^ICC^. [6]

To evaluate the extent to which housing characteristics, pest treatments, sampling characteristics, and nearby agricultural/public land applications explained within-home and between-home variability, we built multivariable mixed-effects models for each of the log-transformed pesticide concentrations. We first constructed models for each imputation data set that added a single potential predictive factor as a fixed-effect term to the null model, which was described previously (Equation 1). Variables that predicted measured pesticide concentrations with *p*-values < 0.25 were candidates for multivariable mixed-effects models. For each pesticide, we fitted an initial model with all candidate variables using each of the five data sets with imputed values below the LOD, combined the results using PROC MIANALYZE, and then removed the variable with the highest *p*-value. We repeated the model fitting process, removing one variable at a time, until all variables had *p*-values ≤ 0.1. The formula for the final model is

ln(*Y_ij_*) = μ*_Y_* + Σβ*X* + *b_i_* + ε*_ij_*, [7]

where *X* represents the final fixed effect variables and β represents the regression coefficients for those fixed effects. We calculated the percentage of each variance component explained by the inclusion of the fixed effects compared with the null model (Egeghy et al. 2005).

## Results

Self-reported pesticide use was common in our study population, with participants reporting at least one type of prior pest treatment at 96% of visits (Table 1). Across all study visits, the most common treatments were for the lawn/garden pests (56% of visits), ants/flies/roaches (47%), and fleas/ticks (37%). The pesticides most commonly applied to crops/public lands within sections located ≤ 1,250 m of the homes were chlorpyrifos (88% of visits), simazine (76%), and diazinon (68%). Approximately 50% of the 21 study homes were built before 1970, and 20% were built during or after 1990. Most homes had a dog (43%) or both a cat and dog (38%), and in all but one of those homes, the animal spent > 1 hr outside per day. In 4 homes (19%), family members routinely removed their shoes prior to entry.

**Table 1:** Frequency of home and garden pest treatments, nearby agricultural and public land use pesticide applications, and housing and sampling characteristics

Characteristic	Frequency [n (%)]
Home and garden pest treatments (n = 68 visits)	
Lawn/garden	38 (56)
Ants/flies/roaches	36 (47)
Fleas/ticks	25 (37)
Professional outdoor only	17 (25)
Professional lawn/yard	13 (19)
Bees/wasps/hornets	11 (16)
Other indoor pests	8 (12)
Professional indoor and outdoor	7 (10)
Any treatment	65 (96)
Agricultural/land use applications to sections within 1,250-m buffer zone (n = 68 visits)a	
Chlorpyrifos	60 (88)
Simazine	52 (76)
Diazinon	46 (68)
Cyfluthrin	31 (46)
Carbaryl	28 (41)
Trifluralin	26 (38)
Permethrin	20 (29)
Cypermethrin	14 (21)
Dacthal	10 (15)
Piperonyl butoxide	2 (3)
Housing characteristics (n = 21 homes)	
Home built before 1970	10 (50)
Home built 1970–1989	6 (30)
Home built 1990 or later	4 (20)
Family member with pesticide-related job	1 (5)
No cat or dog	4 (19)
Owned dog only	9 (43)
Owned both cat and dog	8 (38)
Cat or dog spends > 1 hr outside/day	16 (76)
Shoes routinely removed before entry	4 (19)
Sampling characteristics (n = 68 visits)	
Carpet age < 4 years	22 (32)
Carpet age 4–10 years	22 (32)
Carpet age > 10 years	24 (35)
Collected from living room/family room	55 (81)
Collected from dining room	7 (10)
Collected from bedroom	5 (7)
Collected from hallway	1 (2)
Room of sample collection used as throughway	63 (93)
Months after first study visitb	15 (0–31)
aApplication based on CPUR metric; no applications for chlordane, methoxychlor, propoxur. bThe difference (in months) between the first study visit date and the subsequent visit dates; values reported are median (range).	

Characteristics of the pesticides and their distributions in homes at the first visit (*n* = 21 homes) and all visits (*n =* 68) are shown in Table 2. In general, detection rates and concentrations were similar when comparing the first visit and all visits combined; therefore, here we describe results for the first visit only. Chlordane, a highly persistent insecticide (soil half-life = 350 days) used extensively to treat termites prior to its ban in 1988, had a high frequency of detection (95%). In contrast, methoxychlor, another relatively persistent organochlorine (soil half-life = 120 days) restricted in 2003, had a corresponding lower frequency of detection (48%). The organophosphate insecticides chlorpyrifos and diazinon were commonly detected (100% and 90%, respectively) with similar persistence (soil half-life = 30 days and 40 days, respectively) and were prohibited for residential use prior to the study period (in 2000 and 2002, respectively). We observed relatively higher frequencies of detection (67% and 100%, respectively) for two pyrethroid insecticides with low persistence, cypermethrin and permethrin (soil half-lives = 30 days), whereas another pyrethroid insecticide with the same persistence, cyfluthrin, had a lower frequency of detection (38%). Dacthal, simazine, and trifluralin, all moderately persistent herbicides (soil half-life of 60–100 days), had highly variable frequencies of detection (52–90%).

**Table 2:** Persistence, detection, and distribution of pesticide concentrations in house dust samples, by first visit and all visits.

Pesticide (chemical class)	Year residential use restricted	Soil half-life (days)a	Detection limits (ng/g)	First visit (n = 21)		All visits (n = 68)	
				Percent detected	Median concentration [IQR (ng/g)]	Percent detected	Median concentration [IQR (ng/g)]
Insecticides
Chlordane (organochlorine)	1988	350	2	95	120 (27, 420)	99	100 (24, 290)
Methoxychlor (organochlorine)	2003	120	10	48	ND (ND, 28)	49	ND (ND, 13)
Chlorpyrifos (organophosphate)	2000	30	5	100	48 (29, 120)	100	44 (29, 79)
Diazinon (organophosphate)	2002	40	2	90	24 (11, 120)	94	13 (ND, 34)
Carbaryl (carbamate)	NA	10	2	100	76 (35, 170)	96	43 (26, 110)
Propoxur (carbamate)	NA	30	5	76	33 (12, 72)	79	14 (ND, 33)
Cyfluthrin (pyrethroid)	NA	30	20	38	ND (ND, 470)	46	ND (ND, 380)
Cypermethrin (pyrethroid)	NA	30	20	67	390 (ND, 2,800)	79	340 (110, 660)
Permethrin (pyrethroid)	NA	30	2	100	1,300 (310, 4,000)	100	1,000 (380, 2,500)
Piperonyl butoxide (synergist)	NA	4.3	4	90	280 (95, 1,100)	96	280 (140, 910)
Herbicides
Dacthal (chlorinated benzoic acid)	NA	100	1	52	1.5 (ND, 9.7)	75	1.8 (ND, 3.1)
Simazine (triazine)	NA	60	2	90	34 (12, 85)	96	45 (20, 110)
Trifluralin (dinitroaniline)	NA	60	2	67	4.0 (1.1, 8.3)	84	2.2 (1.4, 4.4)
Abbreviations: IQR, interquartile range; NA, not applicable [the pesticide was not restricted for residential use during the study period (2003–2005) (Agency for Toxic Substance and Disease Registry 1994; U.S. EPA 2004a, 2004b, 2006)]; ND, not detected. aData from Vogue et al. (1994).							

The ICCs for repeated measurements of the pesticides ranged from 0.37 to 0.95 (Table 3). We observed the highest ICCs for chlordane (0.95), permethrin (0.87), and piperonyl butoxide (0.86) and the lowest for simazine (0.37) and carbaryl (0.45). Based on these ICCs, we estimated that using a single pesticide measurement to estimate exposure would result in attenuation bias in the logistic regression coefficient of a hypothetical case–control study ranging from –5 to –63%. We also estimated that if the OR_true_ for an outcome of interest was 2.0, the observed odds ratio would be 1.7–1.9 for 7 of the 13 pesticides. For the remaining 6 pesticides (carbaryl, methoxychlor, chlorpyrifos, cyfluthrin, dacthal, simazine), the attenuation bias was –63 to –31%, yielding observed odds ratios of 1.3 to 1.6, respectively.

**Table 3:** Variance components, ICCs, attenuation bias in logistic regression coefficients, and associated potential attenuation of odds ratios in a hypothetical epidemiologic study.

Chemical class/ pesticide	σ2BW (95% CI)	σ2WI (95% CI)	ICCa	Percent attenuation biasb	ORobs if ORtrue = 2.0c
Carbamates
Carbaryl	1.1 (–0.04, 2.3)	1.3 (0.73, 2.0)	0.45	–55	1.4
Propoxur	2.0 (0.41, 3.6)	0.75 (0.33, 1.2)	0.73	–27	1.7
Organochlorines
Chlordane	3.2 (1.2, 5.1)	0.16 (0.10, 0.23)	0.95	–5	1.9
Methoxychlor	1.8 (–0.31, 3.8)	1.5 (0.36, 2.7)	0.54	–46	1.5
Organophosphates
Chlorpyrifos	0.40 (0.01, 0.79)	0.43 (0.25, 0.61)	0.48	–52	1.4
Diazinon	3.0 (0.47, 5.5)	0.99 (0.46, 1.5)	0.75	–25	1.7
Pyrethroids
Cyfluthrin	1.6 (0.28, 2.8)	0.84 (0.48, 1.2)	0.65	–35	1.6
Cypermethrin	3.6 (0.97, 6.2)	0.74 (0.42, 1.1)	0.83	–17	1.8
Permethrin	2.4 (0.78, 4.0)	0.37 (0.22, 0.53)	0.87	–13	1.8
Synergist
Piperonyl butoxide	6.2 (1.5, 11)	0.99 (0.56, 1.4)	0.86	–14	1.8
Herbicides
Dacthal	2.2 (0.42, 3.9)	0.96 (0.35, 1.6)	0.69	–31	1.6
Simazine	1.3 (–0.36, 2.9)	2.2 (1.2, 3.1)	0.37	–63	1.3
Trifluralin	1.5 (0.44, 2.7)	0.32 (0.10, 0.54)	0.83	–17	1.8
Abbreviations: Abbreviations: σ2BW, between-home variance; σ2W, within-home variance; CI, confidence interval; ICC, intraclass correlation coefficient. aσ2BW/(σ2BW + σ2WI). b[(β1,obs – β1,true)/β1,true] × 100%. cORtrueICC.					

The final mixed-effects models for each pesticide are presented in Table 4. Pest treatment practices, housing characteristics, sampling characteristics, and nearby agricultural/public land applications explained –35 to 44% of the between-home variability and 0 to 39% of the within-home variability in pesticide concentrations. Negative values for percent of variation explained by the models were observed for propoxur, methoxychlor, piperonyl butoxide, and simazine because the magnitude of the between-home variance component from the mixed-effects models was greater than that of the null model. The most between-home variability (29–44%) was explained by mixed-effects models for carbaryl, trifluralin, cyfluthrin, and dacthal. The most within-home variability (27–39%) was explained for piperonyl butoxide, diazinon, and carbaryl.

**Table 4:** Proportional change in pesticide concentration (eβ) and variance components from mixed-effects models and percent of variability explained by the explanatory variables (fixed effects)

Pesticide/ Explanatory variable	Regression coefficients from mixed-effects models (95% CI)	Variance components from null models		Variance components from mixed-effects models	
		σ2BW	σ2WI	σ2BW (% exp)a	σ2WI (% exp)b
Carbamates
Carbaryl		1.10	1.35	0.62 (44)	0.99 (27)
Ants/flies/roachesc	2.25 (1.21, 4.22)
Professional outdoor onlyc	1.21 (0.55, 2.67)
Professional outdoor and indoorc	0.41 (0.15, 1.13)
Months after first visit	0.96 (0.93, 0.98)
Remove shoesd	0.24 (0.08, 0.74)
Propoxur		2.02	0.75	2.14 (–6)	0.58 (23)
Months after first visit	0.96 (0.94, 0.98)
Organochlorines
Chlordane		3.15	0.16	2.66 (16)	0.14 (13)
Home built 1990 or latere	0.13 (0.02, 0.89)
Home built 1970–1989e	0.70 (–0.98, 2.37)
Months after first visit	0.99 (0.97, 1.0)
Methoxychlor		1.76	1.51	2.38 (–35)	1.25 (17)
Professional outdoor onlyc	1.56 (0.52, 4.65)
Professional outdoor and indoorc	0.22 (0.05, 0.94)
Organophosphates
Chlorpyrifos		0.40	0.43	0.32 (20)	0.39 (9)
Bees/wasps/hornetsc	1.86 (1.15, 3.01)
Dog onlyf	2.36 (0.99, 5.61)
Both cat and dogf	1.79 (0.75, 4.25)
Diazinon		2.97	0.99	2.78 (6)	0.65 (34)
Lawn/gardenc	1.73 (1.01, 2.95)
Professional outdoor onlyc	2.97 (1.30, 6.79)
Professional outdoor and indoorc	0.70 (0.20, 2.49)
Months after first visit	0.96 (0.93, 0.98)
Pyrethroids
Cyfluthrin		1.55	0.84	1.08 (30)	0.71 (15)
Professional outdoor onlyc	4.71 (2.03, 10.9)
Professional outdoor and indoorc	1.07 (0.29, 3.02)
Home built 1990 or latere	4.20 (1.06, 16.7)
Home built 1970–1989e	0.54 (0.14, 2.06)
Cypermethrin		3.58	0.74	3.34 (7)	0.60 (19)
Professional outdoor onlyc	3.31 (1.45, 7.53)
Professional outdoor and indoorc	1.40 (0.52, 3.76)
Other indoor pestc	1.42 (0.35, 1.40)
Months after first visit	0.98 (0.95, 1.00)
Permethrin		2.40	0.37	2.36 (2)	0.28 (24)
Professional outdoor onlyc	3.49 (1.75, 6.94)
Professional outdoor and indoorc	1.29 (0.73, 2.26)
Carpet age 4–10 yearsg	3.49 (1.75, 6.94)
Carpet age > 10 yearsg	3.49 (1.75, 6.94)
Synergist
Piperonyl butoxide		6.23	0.99	7.57 (–21)	0.58 (39)
Ants/flies/roachesc	0.39 (0.22, 0.69)
Lawn/gardenc	1.75 (1.03, 2.96)
Professional lawnc	0.31 (0.08, 1.13)
Herbicides
Dacthal		2.20	0.96	1.56 (29)	0.94 (2)
Fleas/ticksc	0.50 (0.23, 1.08)
Dog onlyf	7.63 (1.33, 43.8)
Both cat and dogf	0.28 (–1.42, 1.99)
Simazine		1.30	2.20	1.31 (–1)	1.88 (15)
Ants/flies/roachesc	0.28 (0.12, 0.65)
Trifluralin		1.54	0.32	1.06 (31)	0.32 (0)
CPUR metrich	1.04 (1.01, 1.08)
Abbreviations: σ2BW, between-home variance; σ2W, within-home variance; CI, confidence interval; % exp, percent explained. a[(σ2BWnull–σ2BWmixed-effects)/σ2BWnull] x 100. b[(σ2WInull–σ2WImixed-effects)/σ2WInull] x 100. cReference: no reported treatments of each particular kind of treatment. dReference: did not typically remove shoes before entry. eReference: homes built before 1970. fReference: owned no cat or dog. gReference: carpet < 4 years old. hDensity of agricultural/public land application within a 1,250 m buffer zone around residence.					

Specific pest treatments (e.g., bees/wasps/hornets, professional outdoor pesticide treatments) were predictors (*p* < 0.1) of 10 pesticides. Homes with professional outdoor treatments versus those with no professional treatments had higher concentrations of permethrin, cypermethrin, cyfluthrin, and diazinon. Homes with both professional outdoor and indoor treatments had lower concentrations of carbaryl and methoxychlor compared with homes with no professional treatments. Treatment for ants/flies/roaches was associated with higher concentrations of carbaryl, but lower concentrations of piperonyl butoxide and simazine. Treatment for bees/wasps/hornets was associated with higher concentrations of chlorpyrifos, and lawn/garden pest treatments had higher concentrations of diazinon and piperonyl butoxide. Homes built in 1990 or later had significantly higher levels of cyfluthrin, but lower levels of chlordane, compared with homes built before 1970. Agricultural/public land pesticide application was a significant predictor of trifluralin concentrations. The number of months after the first study visit was associated with decreasing concentrations of carbaryl, propoxur, chlordane, diazinon, and cypermethrin. Compared with having no pets, having a dog only was associated with higher concentrations of chlorpyrifos and dacthal. Homes with sampled carpets either 4–10 or > 10 years old had higher levels of permethrin compared with homes with carpets < 4 years old. Removing shoes before entering the home was associated with lower levels of carbaryl in house dust.

## Discussion

Measurement of pesticides in house dust may be a useful method of exposure assessment because of the ability to analyze numerous pesticide active ingredients and because these measures are independent of participant recall. In this study, we demonstrated relatively high repeatability of several pesticides, adding to the strengths of this exposure assessment approach. For 7 of the 13 pesticides measured in our study population, a single pesticide measurement may be a reasonable surrogate for average exposure over a 2-year period if < 30% attenuation bias in risk estimates is acceptable. In the mixed-effects models, pest treatments, housing characteristics, and sampling characteristics explained up to 43% and 39% of the between- and within-home variability in pesticide concentrations, respectively.

Few studies have investigated temporal variability in pesticide concentration in residential dust. Quirós-Alcalá et al. (2011) measured pesticide concentrations in two house dust samples collected 5–8 days apart from ≤ 26 urban and rural households in California during July to December 2006. Spearman correlation coefficients ranged from 0.78 to 0.92 (*p* < 0.01) for dacthal, chlorpyrifos, permethrin, cypermethrin, and piperonyl butoxide, and diazinon, respectively. Similarly, we observed high correlations (ICC ≥ 0.75) for diazinon, permethrin, cypermethrin, and piperonyl butoxide. However, we observed lower correlations for chlorpyrifos (ICC = 0.48) and dacthal (ICC = 0.69), perhaps because of the longer duration between repeat sample collections in our study (3–15 months). The National Human Exposure Assessment Survey (NHEXAS) conducted in Baltimore, Maryland (Pang et al. 2002) and an Iowa study (Curwin et al. 2005) each observed higher correlations of chlorpyrifos in repeated dust samples (ICC = 0.9 and ICC = 0.6, respectively), compared with our study. Differences in correlations between the present study (2003–2005) and NHEXAS could be because NHEXAS had a shorter time between visits (about 2 months) compared with our study (median of 5 months). The Iowa study measured pesticide concentrations from multiple locations within a home at two time points approximately 4 weeks apart. The higher ICC in that study could also be due to the shorter time between visits.

We considered whether there was a relationship between the ICCs and characteristics of the pesticides (e.g., restricted residential use, persistence, frequency of detection). We did not observe any consistent pattern that could be used *a priori* to predict repeatability. For example, chlorpyrifos and diazinon were commonly detected organophosphates with similar persistence and similar dates of restricted use, but they had very different ICCs (0.48 and 0.75, respectively). The lack of an observed pattern could be partly due to the use of half-life in soil as a proxy for half-life in residential dust and the lack of detailed information on uses of specific active ingredients.

In the mixed-effects models, pest treatments, housing characteristics (e.g., year the home was built, presence of a cat or dog), and sampling characteristics (e.g., months after initial study visit) explained a maximum of 44% and 39% of the between- and within-home variability, respectively. We did not attempt to quantify other sources of variability, such as the variability in sample collection (e.g., relative percent differences of 10–30% between duplicate samples) and in the analytical method. Among the factors we evaluated, the most frequent predictors of pesticide concentrations were self-reported pest treatments in and around the homes, when the home was built, presence of a cat or dog, and months after first study visit. Some important predictors may not have been identified here because of the small sample size and the limited variability for some factors. In addition, some housing characteristics (e.g., shoe removal and presence of a cat or dog) that could have changed over the sampling period were only ascertained at the first visit. Factors that predicted exposure in the opposite direction than expected (e.g., lower concentrations of carbaryl in homes reporting both professional outdoor and indoor treatments compared with no professional treatments) may reflect unmeasured, but correlated, predictors. The negative values for the percent of between-home variance explained observed for four pesticides may reflect the imprecision (i.e., wide confidence intervals) of the estimates of the variance components in the null models, as well as the limited ability of the predictors to provide insights into the variance components for some pesticides.

Few studies have constructed multivariable models of pesticide concentrations in carpet dust in homes without a pesticide-exposed agricultural worker. The largest of these studies, conducted from 1999 through 2001 in Los Angeles County, Detroit, Seattle, and the state of Iowa (Colt et al. 2004), observed higher concentrations of chlordane in older homes, consistent with our study. Colt et al. (2004) also reported significant associations between self-reported pest treatments and dust concentrations of several pesticides, but none of the same pesticide-treatment associations were observed in our population, perhaps because of the differing pest treatment questions, time periods, and geographic regions between the studies. For example, we observed an association between the pyrethroids and professional outdoor treatments, but Colt et al. (2004) did not consider professional treatments as a predictor. Colt et al. (2004) observed higher concentrations of carbaryl in homes with treatment for fleas/ticks and lawn/garden insects, whereas we observed higher concentrations of carbaryl only in homes with treatment for ants/flies/roaches.

Few studies have characterized the percentage of variability explained by pesticide treatments and applications, housing characteristics, and sampling characteristics. An analysis in NHEXAS (Egeghy et al. 2005) investigated numerous potential predictors of within-person and between-person temporal variability of chlorpyrifos in carpet dust, including demographics, housing characteristics, pesticide use, and exposure-related activities. Their model for chlorpyrifos explained 43% and 26% of the between- and within-home variability compared with 20% and 9% in our study, respectively. Although their final model explained more variability, the authors acknowledged that their final model was difficult to interpret. For example, applying pesticides in the bathroom in the prior 6 months was associated with higher chlorpyrifos concentrations, but the number of application days was inversely associated with concentrations, and no association was observed with treatment of other rooms.

The ability of self-reported pest treatments, housing characteristics, and sampling characteristics to explain some of the variability in pesticide dust concentrations suggests that this type of information could be combined with pesticide measurements to improve exposure classification. For example, Colt et al. (2006) used self-reported termite treatments in combination with chlordane (a termiticide) measurements in carpet dust and observed a stronger association with risk of non-Hodgkin lymphoma than when using either exposure assessment method alone.

## Conclusions

Our findings can help inform the design of future epidemiologic studies of pesticide exposure and adverse health outcomes. For the majority of pesticides measured in our study population, a single pesticide measurement may be a reasonable estimate for average exposure over a 2-year period if an attenuation bias of ≤ –30% in risk estimates is acceptable. Further study is needed to determine if our findings hold for longer exposure periods, other geographic regions, and additional pesticides.
